# Supplementary figures and images for: Specific GPCRs elicit unique extracellular vesicle miRNA array signatures
Source: eLife. 2026 Mar 20;14:RP107865. doi: 10.7554/eLife.107865 (PMC13004594; doi:10.7554/eLife.107865)

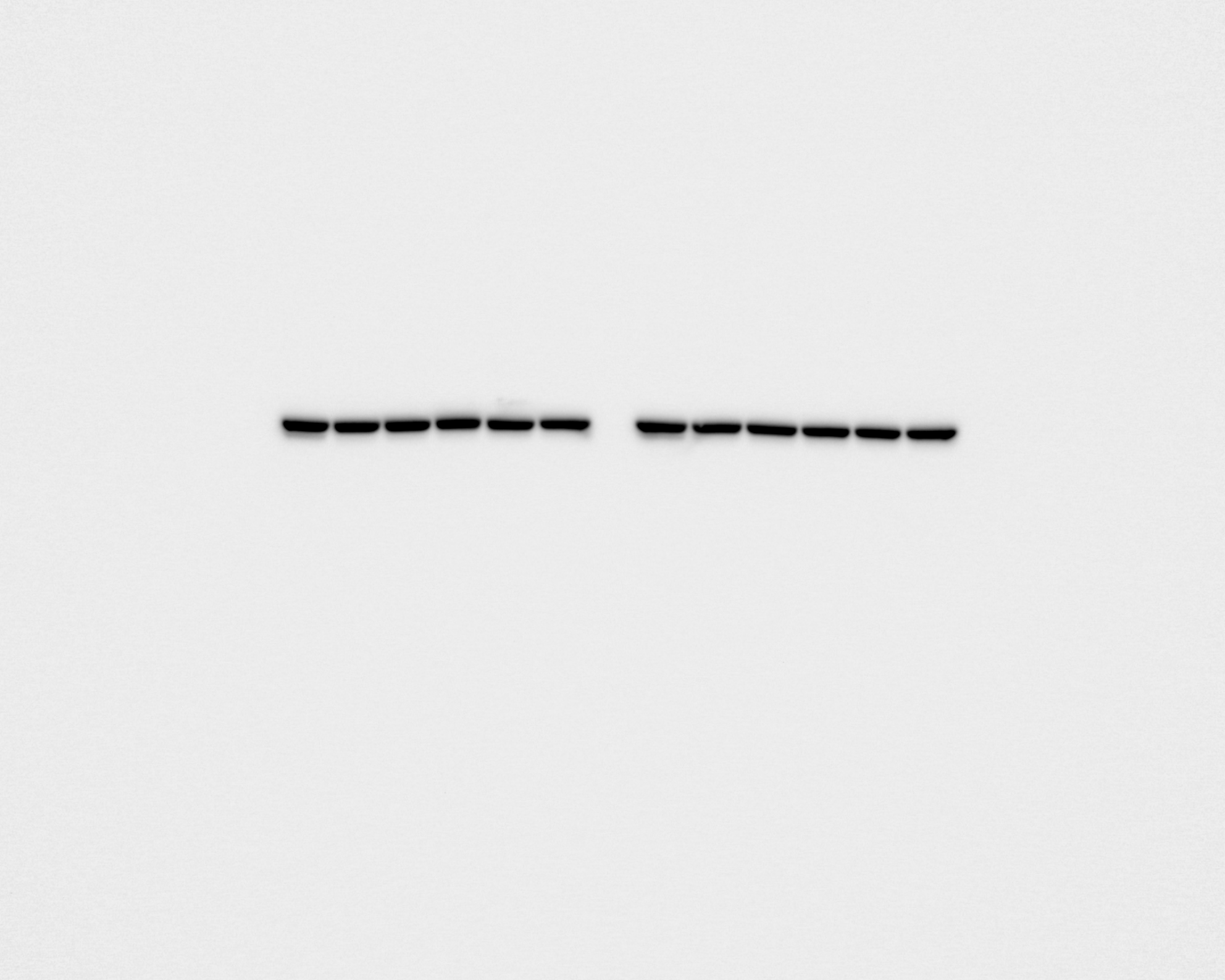

Supplement: Figure 1—figure supplement 1—source data 1. [file elife-107865-fig1-figsupp1-data1.zip › Beta-actin.tif]

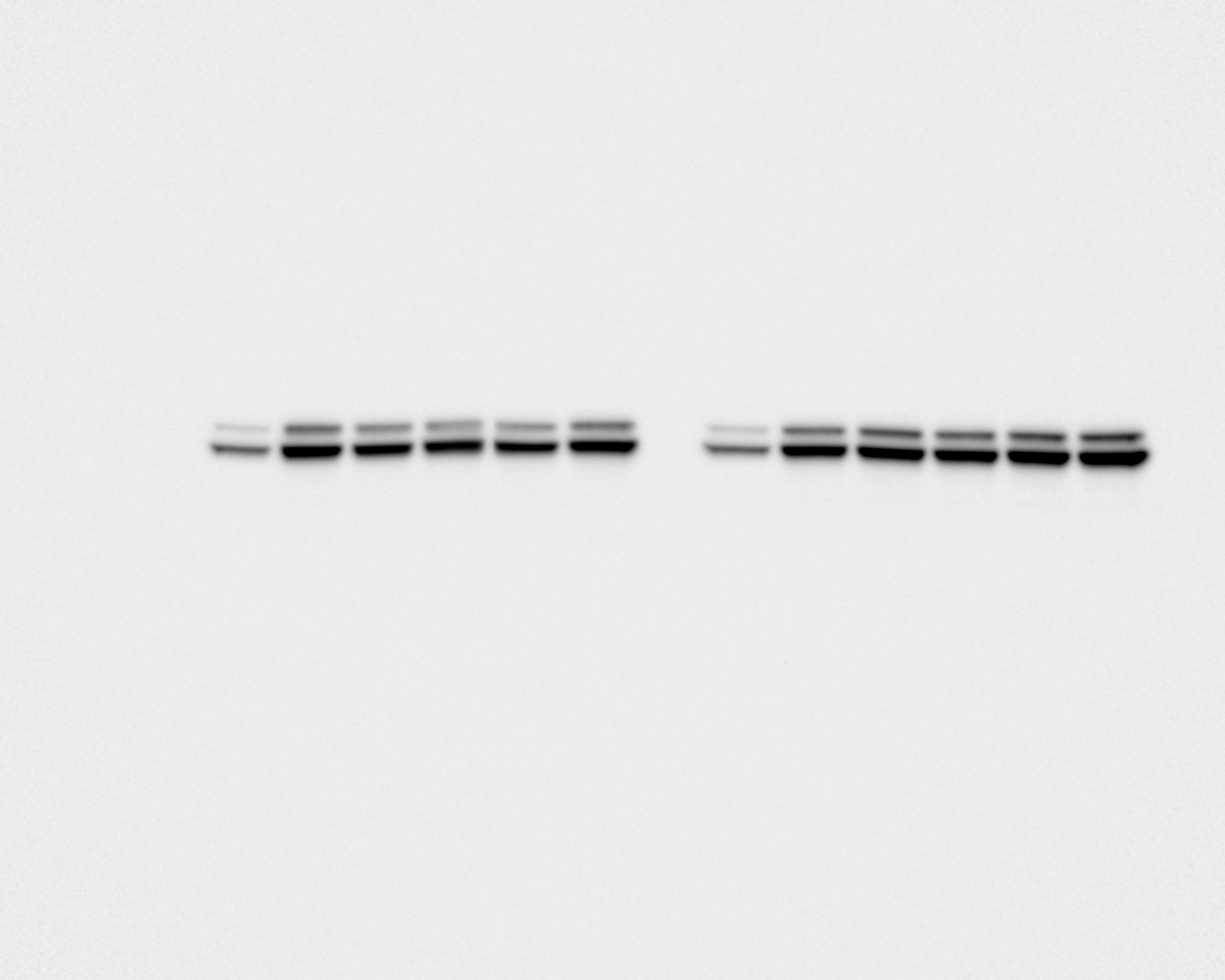

Supplement: Figure 1—figure supplement 1—source data 1. [file elife-107865-fig1-figsupp1-data1.zip › pERK.tif]

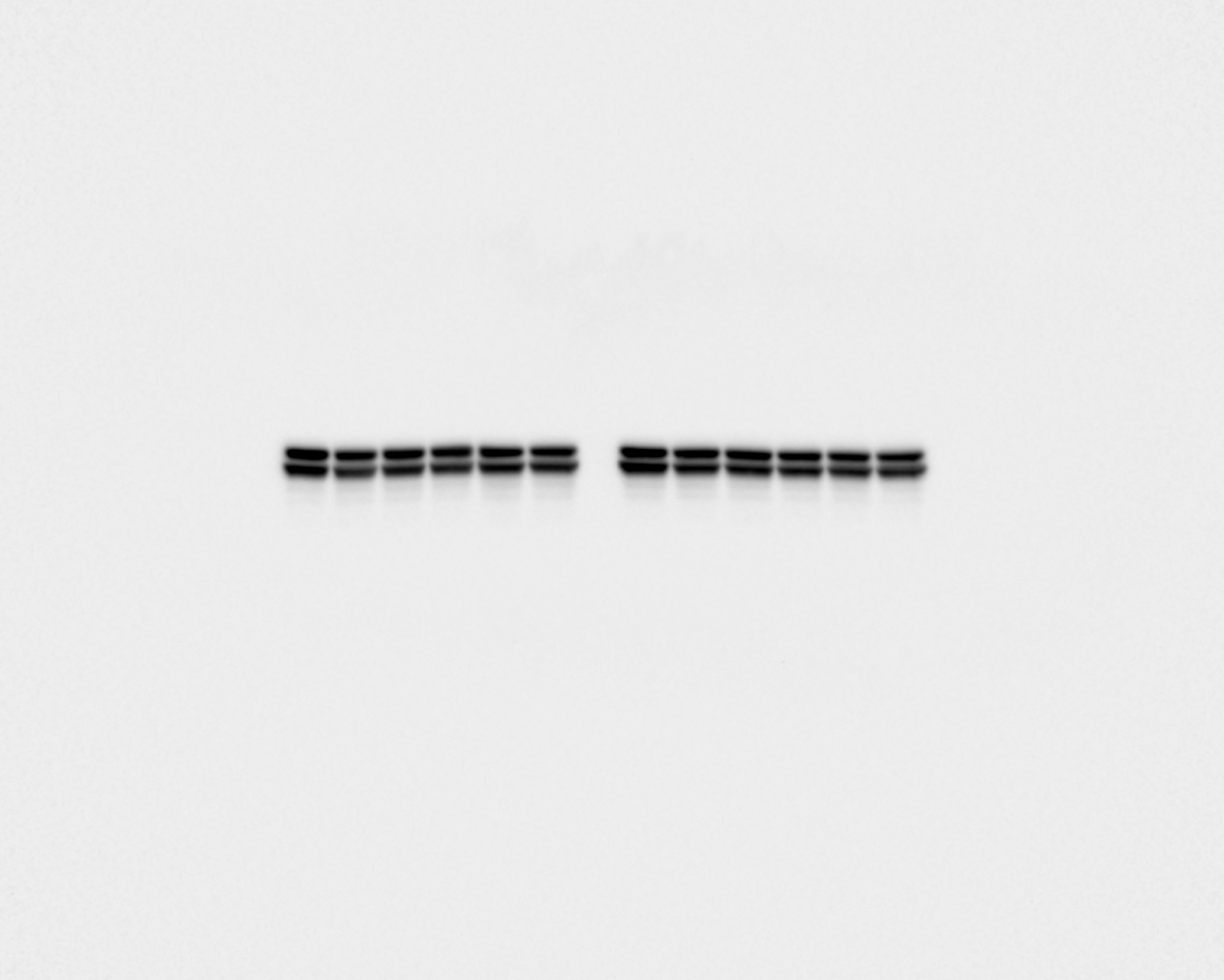

Supplement: Figure 1—figure supplement 1—source data 1. [file elife-107865-fig1-figsupp1-data1.zip › tERK.tif]

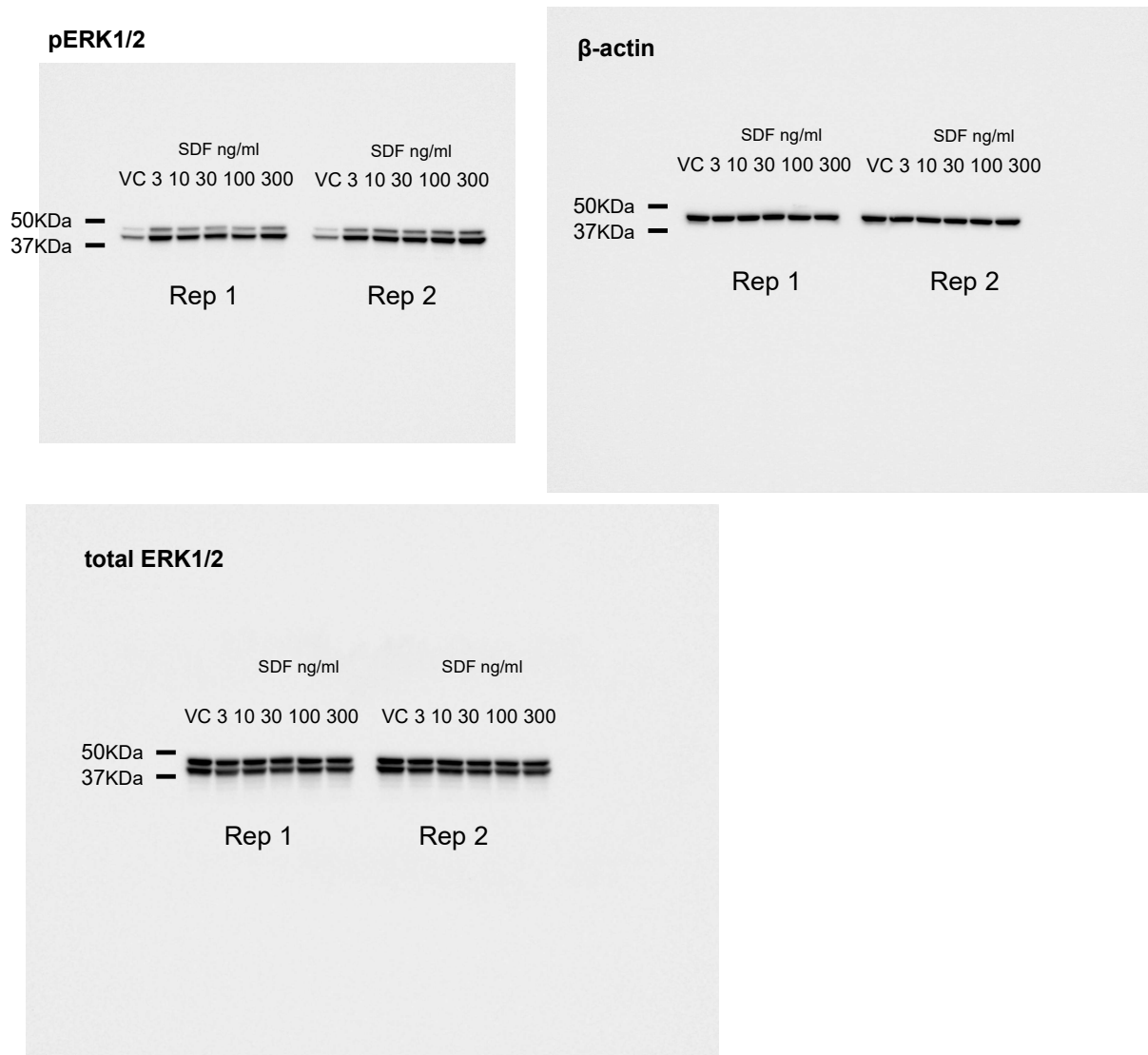

**Figure1-figure supplement 1-Source Data 2.** Original Western blots for Figure1- figure supplement 1.

Supplement: Figure 1—figure supplement 1—source data 2. [file elife-107865-fig1-figsupp1-data2.zip › Figure 1-figure supplement 1-source data 2.pdf]

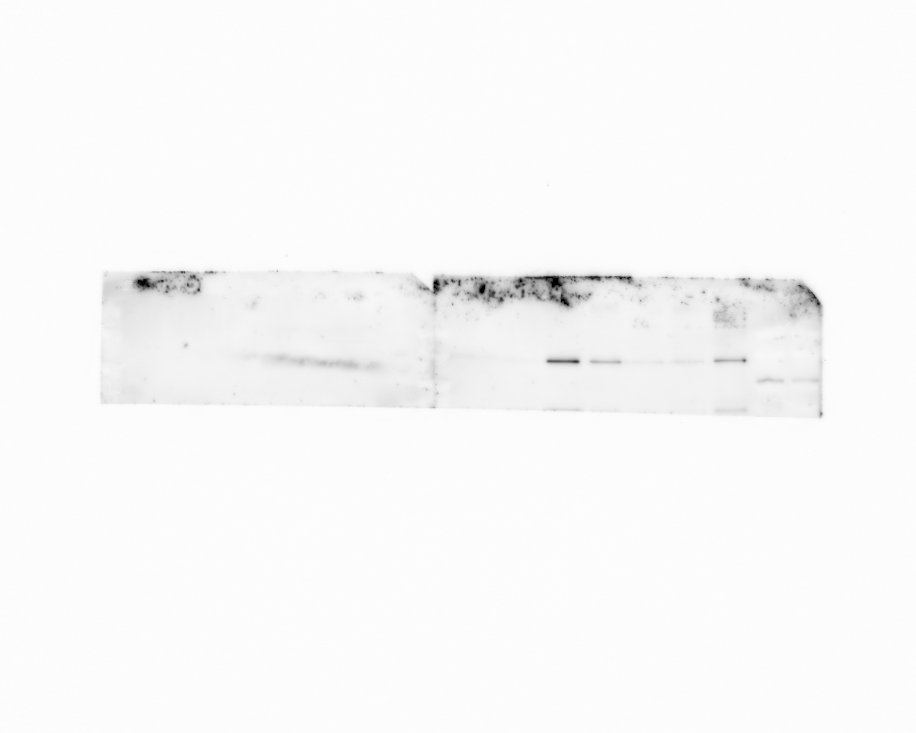

Supplement: Figure 2—source data 1. [file elife-107865-fig2-data1.zip › Calnexin.tif]

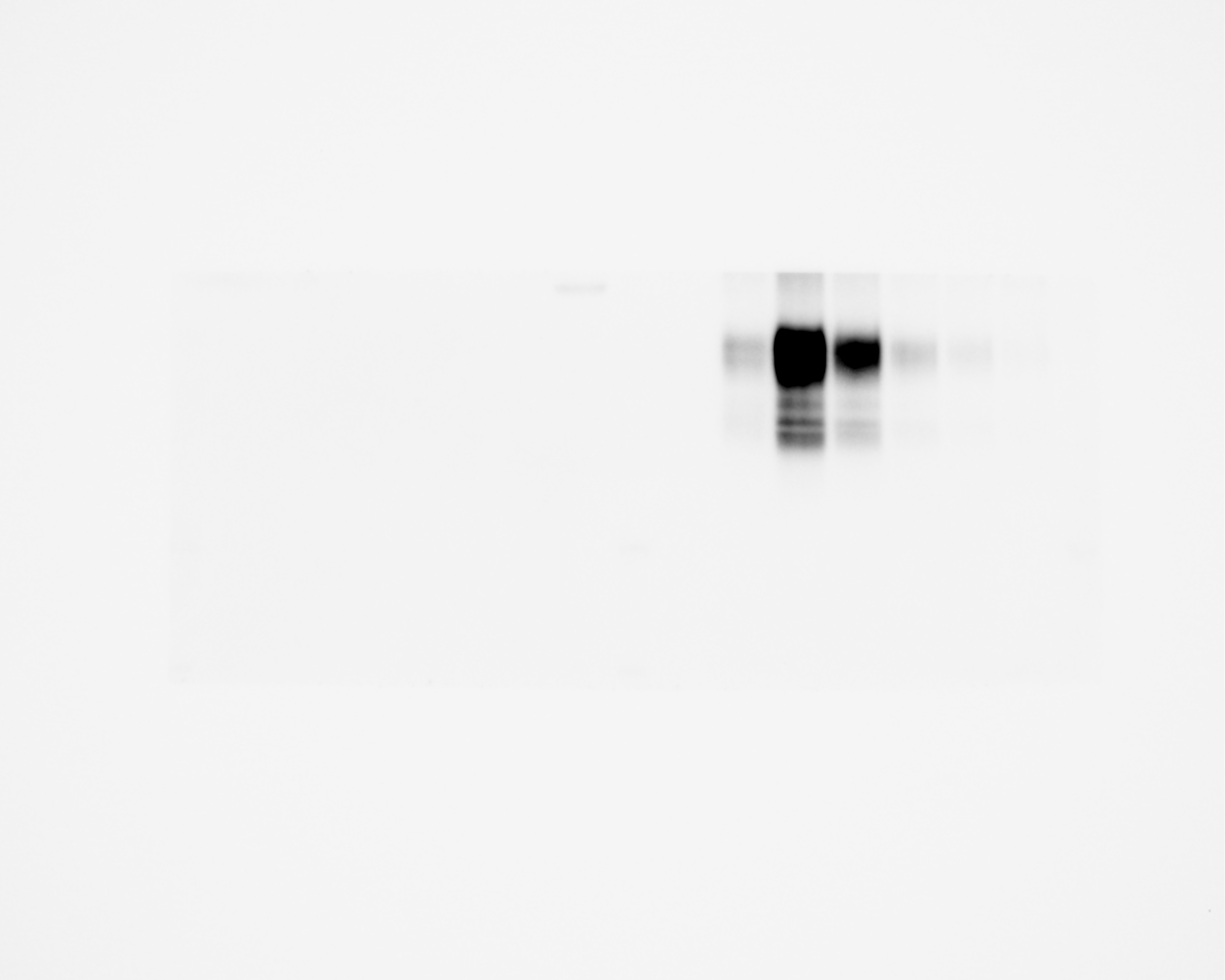

Supplement: Figure 2—source data 1. [file elife-107865-fig2-data1.zip › CD63.tif]

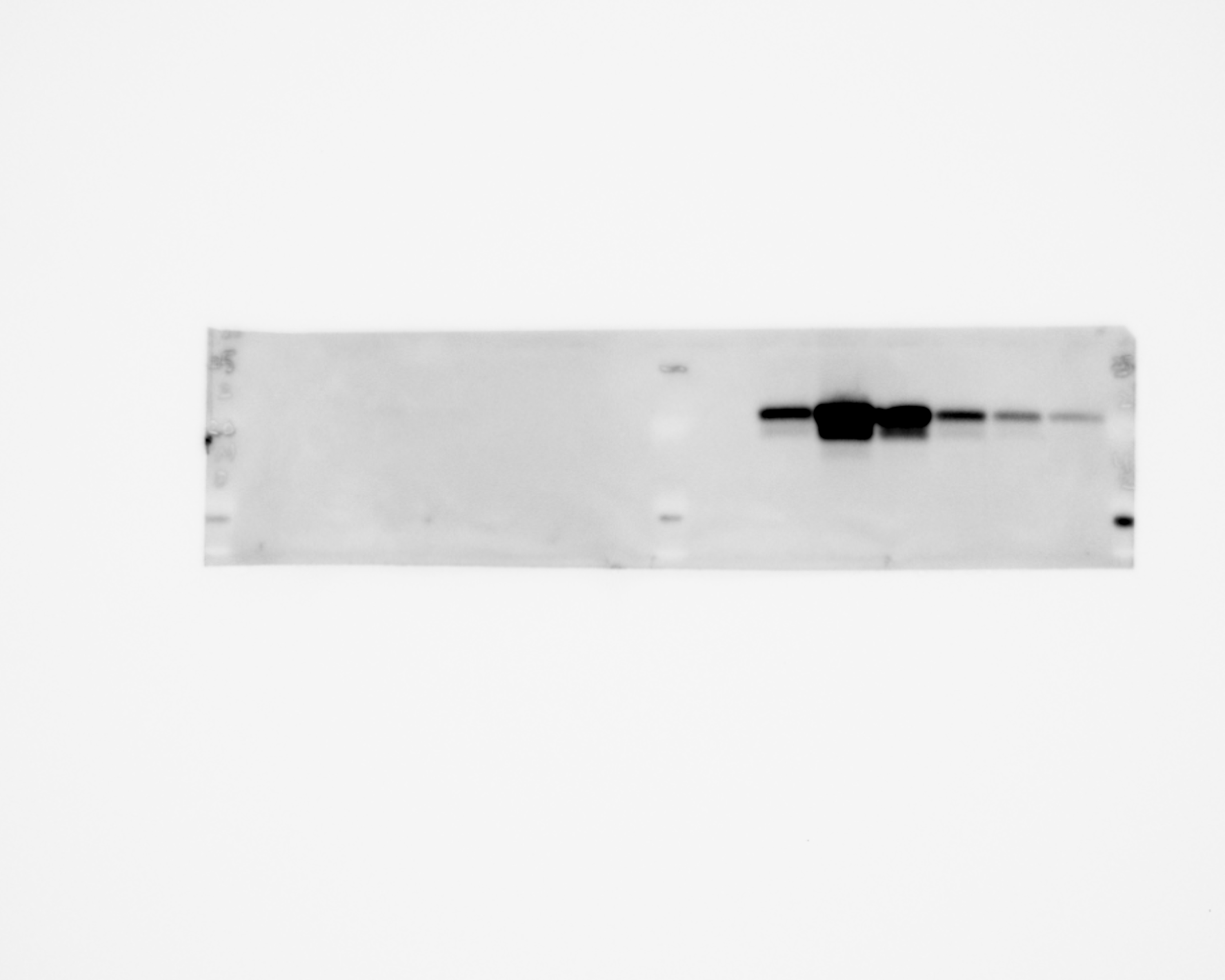

Supplement: Figure 2—source data 1. [file elife-107865-fig2-data1.zip › CD81.tif]

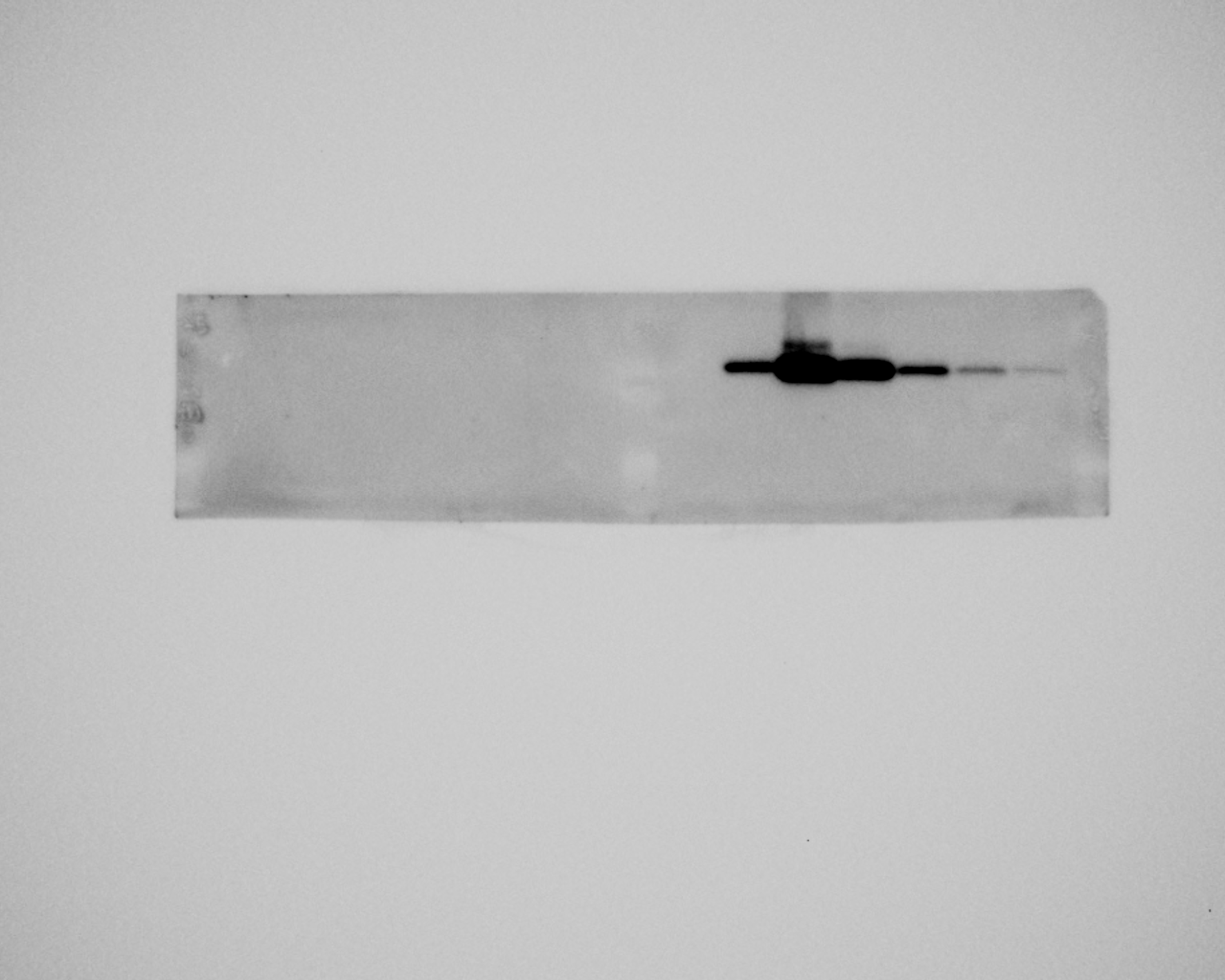

Supplement: Figure 2—source data 1. [file elife-107865-fig2-data1.zip › CD9.tif]

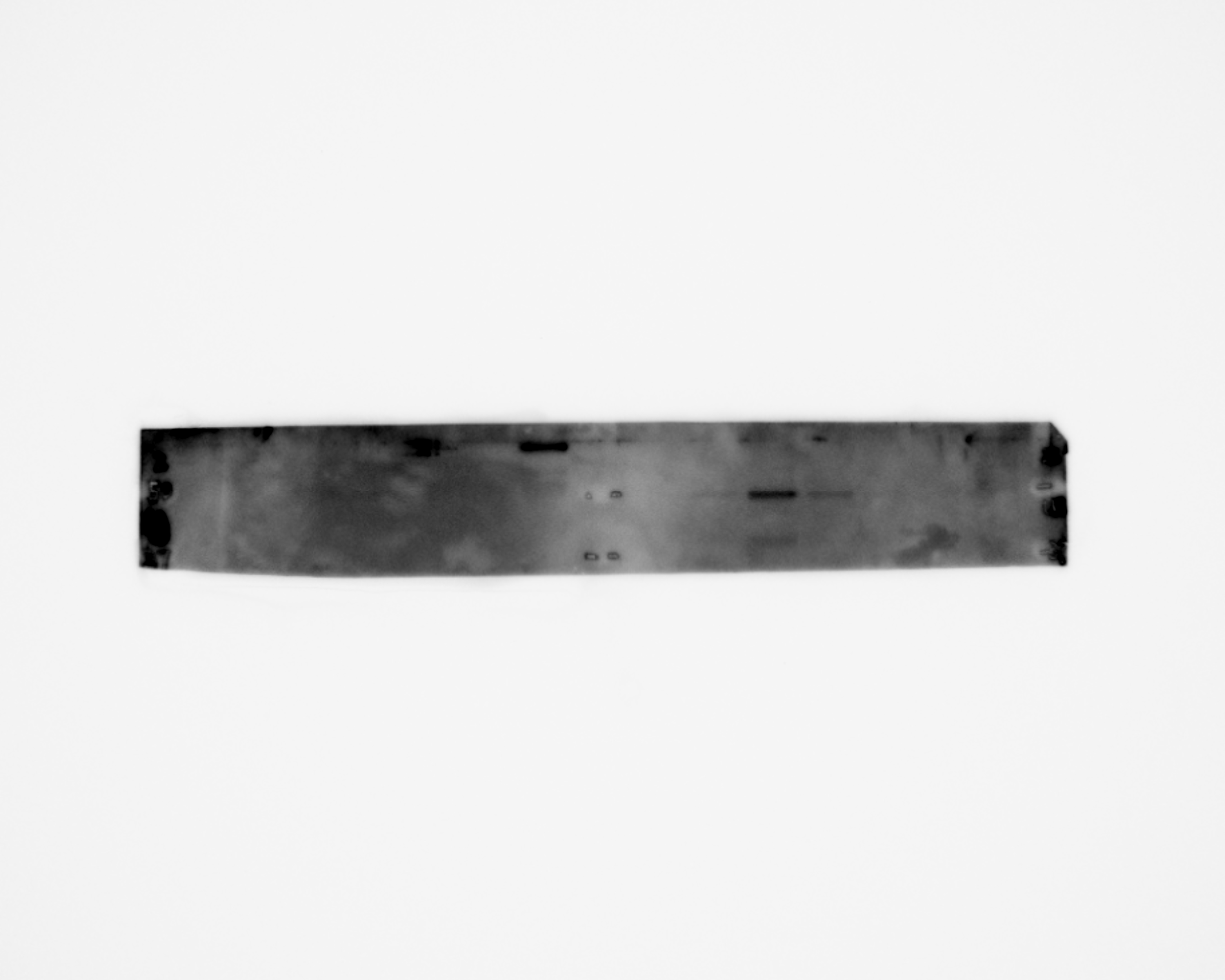

Supplement: Figure 2—source data 1. [file elife-107865-fig2-data1.zip › flotillin.tif]

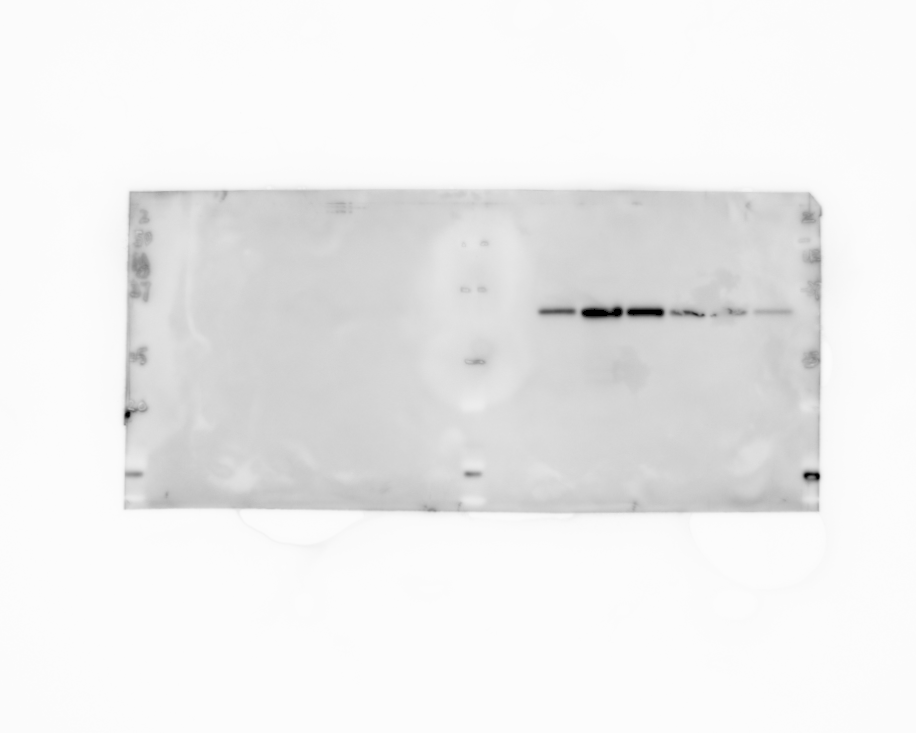

Supplement: Figure 2—source data 1. [file elife-107865-fig2-data1.zip › Syntenin.tif]

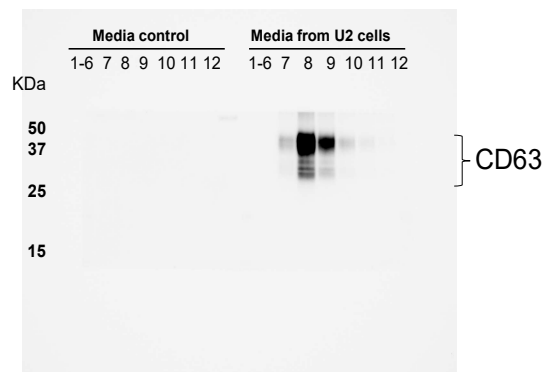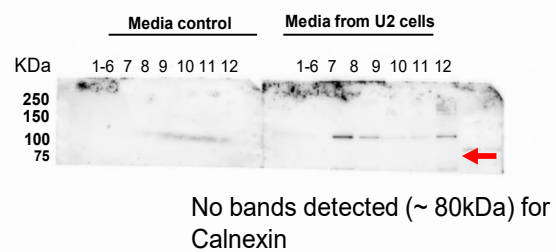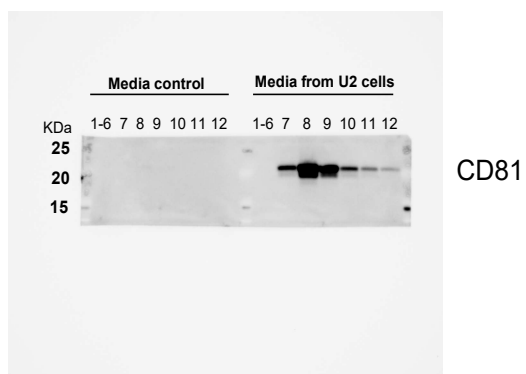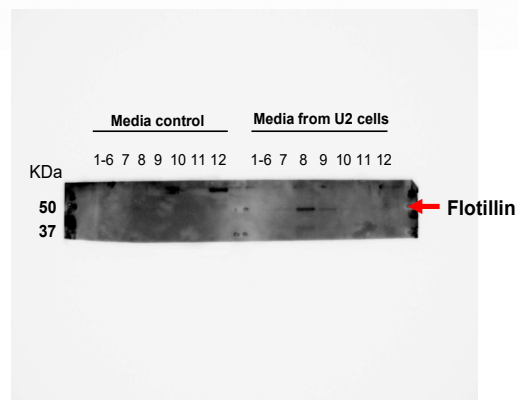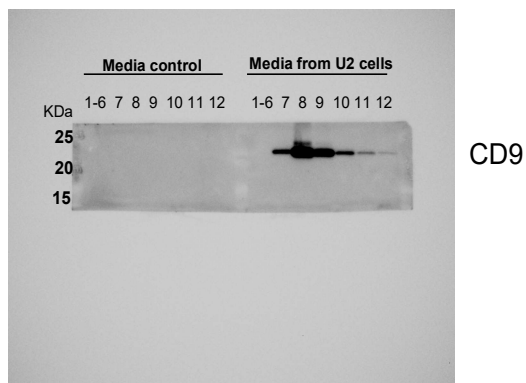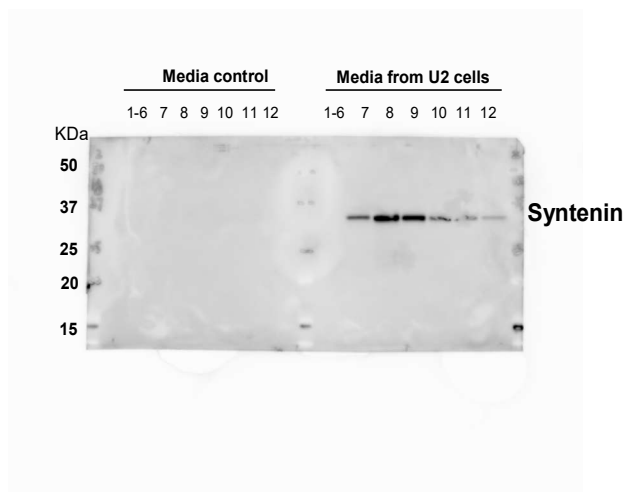

Figure 2-Source data 2. Original Western blots for Figure 2A.

Supplement: Figure 2—source data 2. [file elife-107865-fig2-data2.zip › Figure 2-Source data 2.pdf]
