# Supplementary material for: Specific GPCRs elicit unique extracellular vesicle miRNA array signatures
Source: eLife. 2026 Mar 20;14:RP107865. doi: 10.7554/eLife.107865 (PMC13004594; doi:10.7554/eLife.107865)
Supplement: Supplementary file 1. [file elife-107865-supp1.docx]

| **Cell line** | **Gene** | **Gene description** | **UniProt evidence** |
| --- | --- | --- | --- |
| SH-SY5Y | GRM7 | Glutamate metabotropic receptor 7 | Evidence at protein level |
|  | ADCYAP1R1 | ADCYAP receptor type I | Evidence at protein level |
|  | ADGRA2 | Adhesion G protein-coupled receptor A2 | Evidence at protein level |
|  | ADGRL2 | Adhesion G protein-coupled receptor L2 | Evidence at protein level |
|  | PTGDR | Prostaglandin D2 receptor | Evidence at transcript level |
|  | VN1R1 | Vomeronasal 1 receptor 1 | Evidence at transcript level |
| A-431 | ADGRG2 | Adhesion G protein-coupled receptor G2 | Evidence at protein level |
|  | ADORA2B | Adenosine A2b receptor | Evidence at protein level |
|  | ADRB2 | Adrenoceptor beta 2 | Evidence at protein level |
|  | CELSR2 | Cadherin EGF LAG seven-pass G-type receptor 2 | Evidence at protein level |
|  | EDNRB | Endothelin receptor type B | Evidence at protein level |
|  | FZD3 | Frizzled class receptor 3 | Evidence at protein level |
|  | FZD6 | Frizzled class receptor 6 | Evidence at protein level |
|  | GPR87 | G protein-coupled receptor 87 | Evidence at transcript level |
|  | GRM1 | Glutamate metabotropic receptor 1 | Evidence at protein level |
|  | OR10Q1 | Olfactory receptor family 10 subfamily Q member 1 | Evidence at transcript level |
|  | P2RY2 | Purinergic receptor P2Y2 | Evidence at protein level |
|  | SSTR2 | Somatostatin receptor 2 | Evidence at protein level |
|  | TACR2 | Tachykinin receptor 2 | Evidence at protein level |
| U-251MG | HTR7 | 5-hydroxytryptamine receptor 7 | Evidence at protein level |
|  | S1PR1 | Sphingosine-1-phosphate receptor 1 | Evidence at protein level |
|  | ADRA1B | Adrenoceptor alpha 1B | Evidence at protein level |
|  | CCR4 | C-C motif chemokine receptor 4 | Evidence at protein level |
|  | EDNRA | Endothelin receptor type A | Evidence at protein level |
|  | FZD1 | Frizzled class receptor 1 | Evidence at protein level |
|  | GPR142 | G protein-coupled receptor 142 | Evidence at transcript level |
|  | GPRC5B | G protein-coupled receptor class C group 5 member B | Evidence at transcript level |
|  | LGR6 | Leucine rich repeat containing G protein-coupled receptor 6 | Evidence at protein level |
|  | MAS1L | MAS1 proto-oncogene like, G protein-coupled receptor | Evidence at transcript level |
|  | OR6P1 | Olfactory receptor family 6 subfamily P member 1 | No human protein/transcript evidence |
|  | PTAFR | Platelet activating factor receptor | Evidence at protein level |
| U2OS | GPRC5A | G protein-coupled receptor class C group 5 member A | Evidence at protein level |
|  | GLP2R | Glucagon like peptide 2 receptor | Evidence at protein level |
|  | NMBR | Neuromedin B receptor | Evidence at protein level |
|  | OR4N4 | Olfactory receptor family 4 subfamily N member 4 | Evidence at transcript level |
|  | ACKR2 | Atypical chemokine receptor 2 | Evidence at protein level |
|  | ACKR3 | Atypical chemokine receptor 3 | Evidence at protein level |
|  | ADGRE5 | Adhesion G protein-coupled receptor E5 | Evidence at protein level |
|  | ADGRF4 | Adhesion G protein-coupled receptor F4 | Evidence at protein level |
|  | ADORA1 | Adenosine A1 receptor | Evidence at protein level |
|  | CALCRL | Calcitonin receptor like receptor | Evidence at protein level |
|  | CCR7 | C-C motif chemokine receptor 7 | Evidence at protein level |
|  | CRHR2 | Corticotropin releasing hormone receptor 2 | Evidence at protein level |
|  | DRD4 | Dopamine receptor D4 | Evidence at protein level |
|  | F2RL1 | F2R like trypsin receptor 1 | Evidence at protein level |
|  | FZD4 | Frizzled class receptor 4 | Evidence at protein level |
|  | FZD8 | Frizzled class receptor 8 | Evidence at protein level |
|  | GABBR1 | Gamma-aminobutyric acid type B receptor subunit 1 | Evidence at protein level |
|  | GCGR | Glucagon receptor | Evidence at protein level |
|  | GPR156 | G protein-coupled receptor 156 | Evidence at transcript level |
|  | GPR162 | G protein-coupled receptor 162 | Evidence at protein level |
|  | GPR17 | G protein-coupled receptor 17 | Evidence at protein level |
|  | GPR173 | G protein-coupled receptor 173 | Evidence at protein level |
|  | GPR27 | G protein-coupled receptor 27 | Evidence at transcript level |
|  | GPR34 | G protein-coupled receptor 34 | Evidence at transcript level |
|  | GPR63 | G protein-coupled receptor 63 | Evidence at transcript level |
|  | HRH1 | Histamine receptor H1 | Evidence at protein level |
|  | NPFFR2 | Neuropeptide FF receptor 2 | Evidence at protein level |
|  | S1PR4 | Sphingosine-1-phosphate receptor 4 | Evidence at protein level |
|  | SSTR3 | Somatostatin receptor 3 | Evidence at protein level |
|  | VIPR2 | Vasoactive intestinal peptide receptor 2 | Evidence at protein level |
