# Supplementary material for: Specific GPCRs elicit unique extracellular vesicle miRNA array signatures
Source: eLife. 2026 Mar 20;14:RP107865. doi: 10.7554/eLife.107865 (PMC13004594; doi:10.7554/eLife.107865)
Supplement: Supplementary file 2. [file elife-107865-supp2.docx]

| **miRNA** | **Average Ct** | **SD** |
| --- | --- | --- |
| hsa-miR-106b-5p | 25.7 | 1.4 |
| hsa-miR-126-5p | 27.0 | 0.8 |
| hsa-miR-126-3p | 24.6 | 1.1 |
| hsa-miR-127-3p | 26.4 | 0.7 |
| hsa-miR-1305 | 30.4 | 0.2 |
| hsa-miR-142-3p | 26.4 | 1.6 |
| hsa-miR-150-5p | 26.4 | 0.6 |
| hsa-miR-15b-5p | 27.8 | 1.0 |
| hsa-miR-192-5p | 28.5 | 0.8 |
| hsa-miR-194-5p | 29.6 | 0.3 |
| hsa-miR-204-5p | 28.8 | 0.5 |
| hsa-miR-215-5p | 29.0 | 0.6 |
| hsa-miR-223-3p | 23.1 | 0.9 |
| hsa-miR-25-3p | 28.0 | 0.8 |
| hsa-miR-26a-5p | 26.1 | 0.8 |
| hsa-miR-26b-5p | 29.1 | 1.0 |
| hsa-miR-27b-3p | 30.1 | 0.2 |
| hsa-miR-30b-5p | 25.0 | 0.8 |
| hsa-miR-30c-5p | 25.0 | 1.2 |
| hsa-miR-331-3p | 25.6 | 0.8 |
| hsa-miR-338-5p | 24.5 | 0.1 |
| hsa-miR-376a-3p | 28.7 | 0.5 |
| hsa-miR-378 | 28.8 | 2.6 |
| hsa-miR-409-3p | 26.9 | 1.3 |
| hsa-miR-494-3p | 28.5 | 0.9 |
| hsa-miR-505-5p | 32.3 | 0.8 |
| hsa-miR-520c-3p | 26.3 | 0.2 |
| hsa-miR-520d-3p | 25.8 | 0.9 |
| hsa-miR-539-5p | 29.2 | 1.2 |
| hsa-miR-548a-3p | 30.6 | 0.4 |
| hsa-miR-572 | 26.7 | 0.3 |
| hsa-miR-601 | 29.2 | 0.6 |
| hsa-miR-625-3p | 28.1 | 0.2 |
| hsa-miR-628-5p | 31.1 | 0.6 |
| hsa-miR-875-5p | 32.3 | 0.5 |
| hsa-miR-451a | 24.3 | 1.2 |
