# Supplementary material for: Specific GPCRs elicit unique extracellular vesicle miRNA array signatures
Source: eLife. 2026 Mar 20;14:RP107865. doi: 10.7554/eLife.107865 (PMC13004594; doi:10.7554/eLife.107865)
Supplement: Supplementary file 3. — All differentially expressed miRNAs (meeting p<0.2) after stimulation were listed. [file elife-107865-supp3.docx]

| **miRNA**​ | **Log2 fold change**​ | ***p* value**​ |
| --- | --- | --- |
| **Adenosine A1 receptor (ADORA1)** ​ | | |
| miR-550a-5p​ | 1.7​ | 0.0455​ |
| miR-1227-3p​ | 1.49​ | 0.0253​ |
| miR-454-5p​ | 1.17​ | 0.0143​ |
| miR-340-3p​ | 0.85​ | 0.1797​ |
| miR-95-3p​ | 0.73​ | 0.1024​ |
| miR-31-5p​ | 0.58​ | 0.0143​ |
| miR-592​ | 0.55​ | 0.1797​ |
| miR-100-5p​ | 0.42​ | 0.0253​ |
| let-7d-5p​ | 0.39​ | 0.1024​ |
| miR-99a-5p​ | 0.36​ | 0.0253​ |
| miR-454-3p​ | 0.35​ | 0.1024​ |
| miR-99b-5p​ | 0.31​ | 0.1797​ |
| miR-708-5p​ | 0.28​ | 0.1024​ |
| miR-1291​ | 0.16​ | 0.1797​ |
| miR-92a-3p​ | 0.11​ | 0.1024​ |
| miR-186-5p​ | 0.1​ | 0.0143​ |
| miR-106a-5p​ | 0.1​ | 0.1797​ |
| miR-135b-5p​ | 0.09​ | 0.1024​ |
| miR-27a-3p​ | -0.22​ | 0.1024​ |
| miR-339-3p​ | -0.29​ | 0.1024​ |
| miR-942-5p​ | -0.32​ | 0.1024​ |
| miR-148a-3p​ | -0.33​ | 0.1024​ |
| miR-577​ | -0.33​ | 0.1797​ |
| miR-130a-3p​ | -0.46​ | 0.1024​ |
| miR-769-5p​ | -0.52​ | 0.1024​ |
| miR-361-5p​ | -0.62​ | 0.1797​ |
| miR-1180-3p​ | -0.66​ | 0.1797​ |
| miR-660-5p​ | -0.74​ | 0.1024​ |
| miR-135a-5p​ | -0.79​ | 0.0143​ |
| **Histamine receptor H1 (HRH1)** ​ | | |
| miR-502-3p​ | 1.57​ | 0.0253​ |
| miR-423-5p​ | 0.59​ | 0.0253​ |
| miR-34a-5p​ | 0.57​ | 0.1797​ |
| miR-365a-3p​ | 0.54​ | 0.1024​ |
| miR-519a-3p​ | 0.38​ | 0.1797​ |
| miR-199a-3p​ | 0.37​ | 0.0143​ |
| miR-130a-3p​ | 0.35​ | 0.1024​ |
| miR-99b-5p​ | 0.34​ | 0.1024​ |
| miR-708-5p​ | 0.25​ | 0.1024​ |
| miR-20b-5p​ | 0.18​ | 0.1797​ |
| miR-222-3p​ | 0.16​ | 0.1797​ |
| miR-197-3p​ | -0.11​ | 0.1024​ |
| miR-181a-2-3p​ | -0.18​ | 0.1024​ |
| miR-146a-5p​ | -0.21​ | 0.1024​ |
| miR-146b-5p​ | -0.23​ | 0.1024​ |
| miR-139-3p​ | -0.28​ | 0.0455​ |
| miR-483-5p​ | -0.35​ | 0.1024​ |
| miR-214-5p​ | -0.38​ | 0.1024​ |
| miR-345-5p​ | -0.38​ | 0.1024​ |
| miR-1180-3p​ | -0.42​ | 0.1024​ |
| miR-339-5p​ | -0.54​ | 0.1797​ |
| miR-744-5p​ | -0.62​ | 0.0253​ |
| miR-1291​ | -1.25​ | 0.1024​ |
| **Frizzled class receptor 4 (FZD4)**​ | | |
| miR-518e-3p​ | 1.39​ | 0.0833​ |
| miR-422a​ | 1.2​ | 0.1024​ |
| miR-564​ | 1.1​ | 0.0833​ |
| miR-520e-3p​ | 0.96​ | 0.0833​ |
| miR-320b​ | 0.78​ | 0.1024​ |
| miR-135b-3p​ | 0.72​ | 0.1797​ |
| miR-375-3p​ | 0.48​ | 0.1024​ |
| miR-181a-3p​ | 0.41​ | 0.1797​ |
| miR-577​ | 0.34​ | 0.0143​ |
| miR-1227-3p​ | 0.33​ | 0.1797​ |
| miR-15b-3p​ | 0.15​ | 0.1024​ |
| miR-455-5p​ | 0.15​ | 0.1024​ |
| miR-28-3p​ | -0.09​ | 0.1024​ |
| miR-483-5p​ | -0.46​ | 0.1024​ |
| miR-95-3p​ | -0.5​ | 0.0143​ |
| miR-590-3p​ | -0.5​ | 0.1024​ |
| miR-660-5p​ | -0.51​ | 0.0143​ |
| miR-625-5p​ | -0.51​ | 0.1797​ |
| miR-532-5p​ | -0.69​ | 0.1024​ |
| miR-203a​-3p | -0.9​ | 0.0143​ |
| miR-190a-5p​ | -2.24​ | 0.0833​ |
| miR-137-3p​ | -2.52​ | 0.1797​ |
| **Atypical chemokine receptor 3 (ACKR3)** ​ | | |
| miR-422a​ | 1.11​ | 0.1797​ |
| miR-1255b-5p​ | 0.7​ | 0.1797​ |
| miR-135b-3p​ | 0.67​ | 0.0253​ |
| miR-10b-5p​ | 0.63​ | 0.1797​ |
| miR-516a-3p​ | 0.55​ | 0.1797​ |
| miR-1227-3p​ | 0.49​ | 0.1024​ |
| miR-9-3p​ | 0.37​ | 0.0143​ |
| miR-9-5p​ | 0.32​ | 0.1024​ |
| miR-324-5p​ | 0.3​ | 0.0143​ |
| miR-26a-1-3p​ | 0.29​ | 0.1024​ |
| miR-152-3p​ | 0.28​ | 0.0143​ |
| miR-221-3p​ | 0.26​ | 0.1797​ |
| miR-31-3p​ | 0.26​ | 0.1024​ |
| miR-1180-3p​ | 0.21​ | 0.0833​ |
| miR-708-5p​ | 0.19​ | 0.1024​ |
| miR-125b-1-3p​ | 0.16​ | 0.1797​ |
| miR-590-5p​ | 0.14​ | 0.1024​ |
| miR-339-3p​ | -0.17​ | 0.0143​ |
| miR-191-3p​ | -0.23​ | 0.1024​ |
| miR-125a-5p​ | -0.3​ | 0.0143​ |
| miR-502-3p​ | -0.6​ | 0.1797​ |
| miR-642a-5p​ | -0.7​ | 0.1024​ |
